# Genomic Prediction Strategies for Dry-Down-Related Traits in Maize

**DOI:** 10.3389/fpls.2022.930429

**Published:** 2022-06-30

**Authors:** Pengzun Ni, Mahlet Teka Anche, Yanye Ruan, Dongdong Dang, Nicolas Morales, Lingyue Li, Meiling Liu, Shu Wang, Kelly R. Robbins

**Affiliations:** ^1^Shenyang Key Laboratory of Maize Genomic Selection Breeding, Liaoning Province Research Center of Plant Genetic Engineering Technology, College of Biological Science and Technology, Shenyang Agricultural University, Shenyang, China; ^2^Section of Plant Breeding and Genetics, School of Integrative Plant Sciences, Cornell University, Ithaca, NY, United States; ^3^College of Agronomy, Shenyang Agricultural University, Shenyang, China

**Keywords:** kernel water content, dry-down rate, genomic prediction, MT-GBLUP, correlated traits

## Abstract

For efficient mechanical harvesting, low grain moisture content at harvest time is essential. Dry-down rate (DR), which refers to the reduction in grain moisture content after the plants enter physiological maturity, is one of the main factors affecting the amount of moisture in the kernels. Dry-down rate is estimated using kernel moisture content at physiological maturity and at harvest time; however, measuring kernel water content at physiological maturity, which is sometimes referred as kernel water content at black layer formation (BWC), is time-consuming and resource-demanding. Therefore, inferring BWC from other correlated and easier to measure traits could improve the efficiency of breeding efforts for dry-down-related traits. In this study, multi-trait genomic prediction models were used to estimate genetic correlations between BWC and water content at harvest time (HWC) and flowering time (FT). The results show there is moderate-to-high genetic correlation between the traits (0.24–0.66), which supports the use of multi-trait genomic prediction models. To investigate genomic prediction strategies, several cross-validation scenarios representing possible implementations of genomic prediction were evaluated. The results indicate that, in most scenarios, the use of multi-trait genomic prediction models substantially increases prediction accuracy. Furthermore, the inclusion of historical records for correlated traits can improve prediction accuracy, even when the target trait is not measured on all the plots in the training set.

## Key message

- When data are limited on difficult to measure traits in historical datasets or in sparse phenotyping approaches, the use of correlated traits in multi-trait predictions models significantly increases prediction accuracy.

## Introduction

Maize (*Zea mays* L.) is one of the most widely grown food crop across the world (Lawrence et al., 2008; Shiferaw et al., 2011). With efforts to increase maize grain yield/production to meet the growing global food demand (Ray et al., 2013), mechanization of grain harvesting has become a common practice in many countries (Pari et al., 2020). In maize, low grain moisture content at harvest is essential for efficient mechanical harvesting (Brooking, 1990; Singh et al., 1998; Liu et al., 2020). When the moisture content is low, mechanical harvesting becomes more efficient due to easier grain shelling (Chowdhury and Buchele, 1978), and low grain moisture content at harvest time is highly desirable by farmers as it allows long-term grain storage (Weinberg et al., 2008). Many developed countries have fully implemented mechanical harvesting in maize (Du Plessis, 2003); however, due to differences in technological advancements and climatic conditions, implementation is still limited in many countries (Du Plessis, 2003). In northern China, for example, efficient mechanical harvesting requires grain moisture content of maize hybrids to be between 25 and 40% (Nielsen, 2011), making the reduction in grain moisture at harvest a main objective of maize breeders in China.

Grain moisture content, at harvest time, depends on the dry-down rate at maturity (Liu et al., 2020). Dry-down rate, which refers to the reduction in grain moisture content after physiological maturity, is an important trait for reaching the desired level of grain moisture content at harvest time (Cross, 1985; Cross and Kabir, 1989; Martinez-Feria et al., 2019). Varieties with a fast dry-down rate can stay-green late into the season to provide nutrients to the grain (Arriola et al., 2012) while ensuring lower grain moisture content at harvest. Dry-down rate is a polygenic quantitative trait (Li et al., 2021) and is usually inferred from grain moisture content at physiological maturity and grain moisture content at harvest time (Cross and Kabir, 1989; Kebebe et al., 2015). To determine the physiological maturity of maize, time of black layer formation on the grain, an indication of physiological maturity of the grain (Rench and Shaw, 1971; Daynard, 1972; Carter and Poneleit, 1973), needs to be recorded. Grain moisture content at physiological maturity or at black layer formation is very difficult and time-consuming to measure since it requires diligent monitoring of the grain for black layer formation (Knittle and Burris, 1976; Tekrony and Hunter, 1995). Therefore, predicting grain moisture content at black layer formation from genomic information and readily available correlated trait(s), such as grain moisture content at harvest time (HWC) and flowering time (FT), is desirable and beneficial to drive genetic improvement using multi-trait genome prediction methods (Schulthess et al., 2016).

Genomic selection (GS) is a popular method that implements and improves upon marker-assisted selection (MAS). Genomic selection (GS) is especially beneficial when dealing with complex traits that are affected by many quantitative loci each with very small effects (Goddard and Hayes, 2007; Hayes et al., 2009; Heffner et al., 2009; Jannink et al., 2010; Crossa et al., 2017). Genomic selection (GS) takes advantage of genome-wide molecular markers, single-nucleotide polymorphisms (SNPs), and has been successfully implemented in both animal and plant breeding to predict genomic breeding values (GEBVs) (Ceballos et al., 2015; Hickey et al., 2017; Zenger et al., 2019). Multi-trait genome prediction (MTGP) models have emerged as a promising approach for joint analyses of multiple traits (Guo et al., 2014; Lyra et al., 2017; Lado et al., 2018; Runcie and Cheng, 2019). MTGP benefits from the information of genetically correlated traits in order to improve genomic prediction accuracies for traits that are difficult to measure/record and can be otherwise inferred from readily available correlated traits (Schulthess et al., 2016).

In this study, multi-trait genomic best linear unbiased prediction (MT-GBLUP) models were used to estimate genetic correlations between BWC and HWC and FT. MT-GBLUP was performed using different model training approaches to investigate optimal prediction strategies and investigate prediction accuracy for BWC when using HWC and FT as secondary traits.

## Materials and Methods

### Materials

The population used in this study contained 397 diverse maize inbred lines with a wide genetic background. These lines were sourced from China (281 lines), United States (105 lines), and CIMMYT (11 lines). Most of the inbred lines from the United States and China are from a temperate environment background, whereas the inbred lines from CIMMYT are from tropical backgrounds.

**FIGURE 1:**
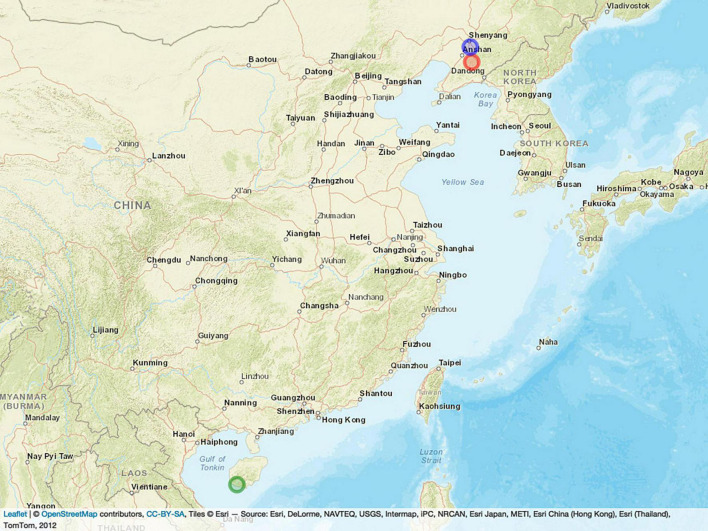
Locations of the three field trials. The blue, red, and green circles represent SN, SY, and HN, respectively.

All 397 inbred lines were planted in three locations in China: Shenyang City in 2019 (19SN) located in northeastern China (N40°82’, E123°56’), Shenfu City in 2017 (17SF) located in northeastern China (N41°51’, E123°54’), and Hainan Province in 2017 (17HN) located in southern China (N18°45’, E109°10’). Figure 1 shows the location of the three field trials in the experiment, where the blue, red, and green circles represent SN, SY, and HN, respectively. All lines were planted using a randomized complete block design with two replicates per line. The lines were planted in a single row plot of 2-m long, 0.6-m wide, with a 0.4-m aisle between rows.

Since grain moisture at physiological maturity is a component of dry-down rate calculations (Cross and Kabir, 1989; Yang et al., 2010), it was important to determine when the inbred lines entered maturity. Using black layer as a mark for maturity (Daynard and Duncan, 1969; Daynard, 1972; Carter and Poneleit, 1973), all maize inbred lines were phenotyped for time to black layer formation. This was done by observing all plants after pollination until the starch layer of maize grains gradually decreased and the black layer formed. When the black layer appeared, the water content of the kernels was measured for six plants that were randomly selected from each inbred line and showed uniform growth. The water content of the kernels was measured with a moisture meter to a depth of 3 mm at two time points, first when the black layer appeared, and then either 15 or 7 days, for temperate or tropical, respectively. Hainan province is located in the tropical zone with little rainfall and high temperatures, so the dry-down rate of kernels is faster than in temperate zones. A preliminary experiment was done and found that approximately 90% of the lines were ready for harvest after 15 and 7 days after physiological maturity in temperate or tropical zones, respectively. Therefore, a modification to dry-down rate was made to ensure correct comparisons between tropical and temperate zones, after the black layer formation as the moisture content at harvest. Days to flowering was defined as the time taken from planting for 50% of the plants in a plot to commence flowering. The experiment was conducted in two ecological zones, the temperate and tropical. The temperate ecological zone had two locations (SN and SF), while the tropical ecological zone had only one location (HN). All phenotypes were collected on the 397 inbred lines.

Genotyping-by-sequencing data with 600 K single-nucleotide polymorphism (SNP) markers were available for the 397 inbred lines. After quality control for missing rate (< 10%), minor allele frequency (MAF > 0.05), and LD pruning (at 0.9), missing genotypes were imputed using TASSEL 5.0 (Bradbury et al., 2007).^1^ A total of 56,563 SNP markers were used.

### Methods

Dry-down rate was calculated for the temperate and tropical regions as follows in Equations 1 and 2.


(1) $$D\,R_{T\,R\,O}=\frac{\left(B\,W\,C-H\,W\,C\right)}{7}$$

(2) $$D\,R_{T\,E\,M}=\frac{\left(B\,W\,C-H\,W\,C\right)}{15}$$

where *DR*_*TRO*_ and *DR*_*TEM*_ are dry-down rate (DR) for the tropical and temperate climate zone, respectively. BWC is grain moisture content when black layer appeared, and HWC is grain moisture content at harvest time.

Single-trait genomic best linear unbiased predictions (ST-GBLUP) were used to estimate genetic and residual variances in each location using the following model for each trait (BWC, HWC, and FT):


(3) y=μ+Xb+Zu+e

where **y** is the vector of raw phenotypes, **μ** is the overall mean, **b** is the fixed effect of replication, **u** is the vector of random additive genetic effects for inbred lines, **X** is a design matrix for the fixed effect of replicate, **Z** is the design matrix for additive genetic effects, and **e** is the vector of residuals. The distribution of the random effect **u** was assumed to be $\text{u}∼\text{N}\,\left(0,\sigma_{u}^{2}\,⊗\text{G}\right)$, where $\sigma_{u}^{2}$ is the additive genetic variance and the **G** is the additive genomic relationship matrix between the inbred lines (Vanraden, 2008) calculated as follows:


(4) $$\text{G}=\frac{\text{WW}{}^{\prime }}{2\,\sum p_{j}\,\left(1-p_{j}\right)}$$

Elements of matrix **W** are *w*_*ij*_ where *w*_*ij*_ is the genotype represented as the number of copies of the major allele of line *i* at marker *j*, denoted as 0 or 2 for the minor and major homozygous genotypes, respectively, and *p_j_* is the allele frequency at marker *j*. Each column of **W** is mean centered prior to calculation of **G**.

Narrow sense heritability (*h*^2^) explains the proportion of phenotypic variation due to additive genetic variance. Heritability was calculated as follows:


(5) $$h^{2}=\frac{\sigma_{u}^{2}}{\sigma_{u}^{2}+\sigma_{e}^{2}}$$

Where $\sigma_{u}^{2}$ is the additive genetic variance, and $\sigma_{e}^{2}$ is the residual error. Variance components were estimated by fitting a ST-GBLUP model with the genomic relationship matrix (GRM).

A MT-GBLUP model was fit to estimate the genetic and residual covariance between three traits: black layer water content (BWC), harvest time water content (HWC), and flowering time (FT). The general MT-GBLUP model within each ecological zone was as follows:


(6) $$\left[\begin{array}{c} {\text{y}}_{1}\\ {\text{y}}_{2}\\ {\text{y}}_{3} \end{array}\right]=\begin{array}{c} \mu_{1}\\ \mu_{2}\\ \mu_{3} \end{array}+\left[\begin{array}{ccc} {\text{X}}_{1} & 0 & 0\\ 0 & {\text{X}}_{2} & 0\\ 0 & 0 & {\text{X}}_{3} \end{array}\right]\,\left[\begin{array}{c} {\text{b}}_{1}\\ {\text{b}}_{2}\\ {\text{b}}_{3} \end{array}\right]+\left[\begin{array}{ccc} {\text{Z}}_{1} & 0 & 0\\ 0 & {\text{Z}}_{2} & 0\\ 0 & 0 & {\text{Z}}_{3} \end{array}\right]\,\left[\begin{array}{c} {\text{u}}_{1}\\ {\text{u}}_{2}\\ {\text{u}}_{3} \end{array}\right]+\begin{array}{c} {\text{e}}_{1}\\ {\text{e}}_{2}\\ {\text{e}}_{3} \end{array}$$

where **y_1_**, **y_2_**, and **y_3_** are the vectors of phenotypes for BWC, HWC, and FT, respectively, **μ_1_**, **μ_2_**, and **μ_3_** are the overall mean for each trait, **b_1_**, **b_2_**, and **b_3_** are the fixed effects of location and replication nested within location, **u_1_**,**u_2_**, and **u_3_** are vectors of the random additive genetic effects for each trait, **X_1_**, **X_2_**, and **X_3_** are the design matrices for the fixed effect of replication, **Z_1_**, **Z_2_**, and **Z_3_** are the design matrices for the random genetic effect, and **e_1_**, **e_2_**, and**e_3_** are the vectors of residuals. It was assumed that [**u_1_**, **u_2_**, **u_3_**] ∼ **N**(0,**G_o_**⊗**G**), where **G**_o_ is the variance–covariance matrix of the genetic effect of the traits as follows:


(7) $${\text{G}}_{0}=\left[\begin{array}{ccc} \sigma_{g\,1}^{2} & \sigma_{g\,12} & \sigma_{g\,13}\\ \sigma_{g\,21} & \sigma_{g\,2}^{2} & \sigma_{g\,23}\\ \sigma_{g\,31} & \sigma_{g\,32} & \sigma_{g\,3}^{2} \end{array}\right]$$

where **G_0_** represents a symmetrical 3 × 3 variance–covariance matrix of the genomic effect of genotypes in the environments. The diagonal of the **G_0_** matrix is the additive genetic variance for three traits, while the off-diagonal elements represent the genetic covariance between the traits.

**G** is the same as Eq. 3, and residual errors were assumed to be distributed as [**e_1_**, **e_2_**, **e_3_**] ∼ **N**(0,**I**⊗**R**), where **I** is the identity matrix and **R** is a symmetrical unstructured matrix of the residual (co) variances:


(8) $$\text{R}=\left[\begin{array}{ccc} \sigma_{1}^{2} & \sigma_{12} & \sigma_{13}\\ \sigma_{21} & \sigma_{2}^{2} & \sigma_{23}\\ \sigma_{31} & \sigma_{32} & \sigma_{3}^{2} \end{array}\right]$$

ST-GBLUP and MT-GBLUP models were fit separately for each ecological region. For the temperate zone where there were two locations, both Eqs 3 and 6 were modified to account for the random location effect and the effect of replicate was nested within location. However, since there is only one location in the tropical ecological zone, the location effect in both Eqs 3 and 6 was ignored.

### Multi-Trait Genomic Prediction and Cross-Validations

Genomic predictions were performed using MT-GBLUP and ST-GBLUP using Eqs 6 and 3 for different cross-validation scenarios. This was done to assess prediction accuracy for BWC using information on correlated traits, HWC and FT, in each ecological zone. For that purpose, three cross-validation scenarios were considered.

**FIGURE 2:**
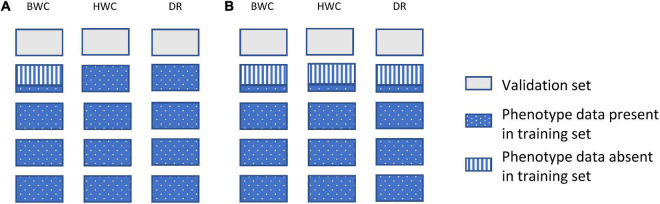
Data used for ST-GBLUP and MT-GBLUP prediction in CV1. Each box indicates the presence or absence of phenotypic data for a particular trait in either the training or validation set. The presence and absence of phenotypic data are indicated by blue dotted (phenotypic data present in the training set), gray (phenotypic data absent in the validation set), and blue vertical stripes (phenotypic data absent in the training set). The phenotypic information for HWC and FT was either kept as complete in the training set **(A)** or set to missing when BWC was missing **(B)**.

Figure 2 shows an example of the first cross-validation scenario (CV1). In CV1, a standard 5-fold cross-validation scenario was used; however, the phenotypic value for BWC was set to missing for an additional randomly selected 20, 40, or 60% of the training set. The phenotypic information for HWC and FT was either kept as complete (Figure 2A) or set to missing (Figure 2B) when BWC was missing. The purpose of this scenario was to examine a genomic selection approach in which historical data are used to predict performance of untested lines and to determine the value of including historical records for correlated traits, even when the target trait (BWC) was missing. This represents a likely scenario as the cost of phenotyping BWC on all tested lines at all test locations will likely be cost prohibitive.

**FIGURE 3:**
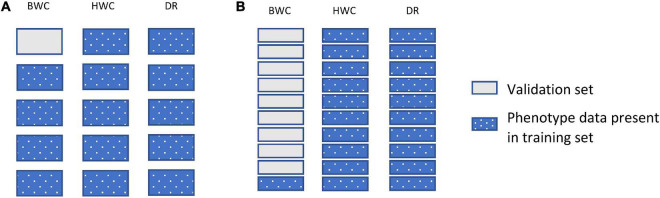
Data used for ST-GBLUP and MT_GBLUP prediction modeling in CV2 **(A)** and the CV_90 scenario where 90% of the inbred lines were randomly selected and had BWC phenotypes set to missing **(B)**. Each box indicates the presence or absence of the phenotypic data for a particular trait on either the training or validation set. The presence and absence of phenotypic data are indicated by dotted blue and gray filled, respectively.

Figure 3 shows an example of the second cross-validation scenario (CV2). CV2 tested prediction accuracy using 10-, 5-, 3-, and 2-fold cross-validation. In each case, the entire dataset was subdivided to groups, with one of the groups used as a validation set (BWC set to missing), with the rest of the group used as a training set. Figure 3B shows an additional scenario (CV_90), in which the validation set was constructed by setting the BWC phenotype of 90% of the maize inbred lines to missing. In this scenario, 90% of the population were randomly selected and BWC was set to missing and fit using MT-GBLUP model. This process was replicated 10 times. In all CV2 scenarios, the phenotypic value for BWC was set to missing in the validation set, while keeping the phenotypic information for HWC and FT. This scenario was used to compare the prediction accuracy in a sparse phenotyping scenario where only a subset of lines are phenotyped for the difficult to measure BWC trait. To examine the impact of using genomic information on prediction accuracy, as opposed to only using correlated trait information, the MT-BLUP model was also fit with an identity matrix in place of the GRM.

In the third scenario (CV3), the validation and training sets were constructed in such a way that one replication or one location was selected and used as a validation set, with the remaining locations and replications used as a training set. In the validation set, the phenotypic value for BWC was set to missing while keeping phenotypic information for HWC and FT. The purpose of this scenario was to simulate a breeding program where BWC is measured only in one replicate or in one location while HWC and FT are recorded in all replicates and locations.

The prediction models were run 10 times, and the Pearson correlation between phenotypic values for BWC (corrected for fixed effects) and predicted values was calculated in each run. The result presented here is the average of the 10 runs. All single- and multi-trait analyses were done using ASReml 4 (Gilmour, 1997)^2^.

## Results

### Heritability and Genetic Correlations

Heritability estimates for BWC, HWC, and DR were obtained using the ST-GBLUP model. As shown in Table 1, small (0.22)-to-moderate (0.69) heritability estimates were obtained for BWC across the different locations and ecological zones. Heritability estimates for HWC ranged from small (0.27) to moderate (0.51) across locations and ecological zones, and heritability estimates for DR ranged from 0.15 to 0.26. Table 2 shows MT-GBLUP genetic correlations, genetic variance, and genetic covariance between BWC, HWC, and FT in temperate ecological zone. Genetic correlations between the BWC, HWC, and FT ranged from 0.24 to 0.66, with the highest genetic correlation between BWC and HWC and the lowest between BWC and FT. Low-to-moderate heritability estimates for BWC and HWC indicate that effective selection pressure can be placed on these traits, and high genetic correlations between BWC, HWC, and FT suggest that multi-trait genomic selection may represent the best approach for genomic prediction for these traits.

**TABLE 1:** ST-GBLUP heritability estimates for BWC, HWC, and DR within each agro-ecological zone and location.

Ecological zone	Location	Traits		
		
		BWC	HWC	DR
Temperate	Shenfu	0.45	0.47	0.22
	Shenyang	0.25	0.27	0.15
	Combined locations	0.22	0.28	0.18
Tropical	Hainan	0.69	0.51	0.26

**TABLE 2:** MT-GBLUP genetic correlation, genetic variance, and genetic covariance between BWC, HWC, and FT in the temperate ecological zone.

	BWC	HWC	FT
BWC	11.59	0.66	0.24
HWC	4.36	3.78	0.42
FT	4.42	4.29	28.16

*Genetic variance of the traits is presented on the diagonal; the upper diagonal shows the genetic correlation between the traits, and the lower diagonal is the genetic covariance between the traits.*

### Prediction Accuracies

**FIGURE 4:**
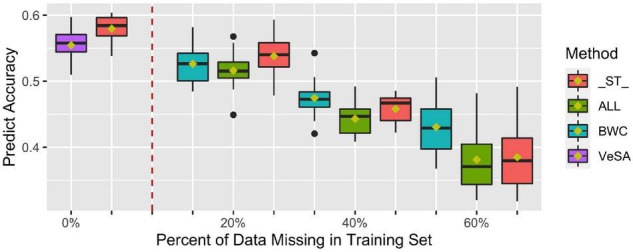
Prediction accuracy from ST-GBLUP and MT-GBLUP models from the first cross-validation (CV1) scenario in temperate zone. Method “_ST_” is the ST-GBLUP model; Method “ALL” is a multi-trait model with all the three dry-down-related traits set to missing for an additional 20, 40, and 60% of the training set; Method “BWC” is a multi-trait model where only the phenotype for BWC is missing for an additional 20, 40, and 60% of the training set; Method “VeSA” is a multi-trait model with complete phenotypic data for all traits in the training set. The results to the left of the dashed lined had no missing data for any trait in the training set.

Figure 4 shows prediction accuracy for BWC in the temperate ecological zone from CV1. Only results from the temperate zone (2 locations) are illustrated, as inconsistent model convergence was observed for the tropical environment, likely due to the limited phenotypic data collected in tropical zone. The box plots on the left side of the dotted line are prediction accuracies where all individuals in the training set have phenotypes for the three traits (BWC, HWC, and FT) and when 5-fold cross-validation without additional missing data in training set was used (purple bars). As shown in the Figure 4, when all the three dry-down-related traits were set to missing for additional 20, 40, and 60% of the training set (green bars), lower prediction accuracies were observed compared to the case where only the phenotype for BWC was set to missing (blue bars). ST-GBLUP model gave the highest accuracy (red bars) in the cases where the phenotype for BWC was set to missing for additional 20% of validation set, but the MT-GBLUP model performed best when all correlated phenotypes were included in the training set and BWC was set to 40 and 60% missing.

**FIGURE 5:**
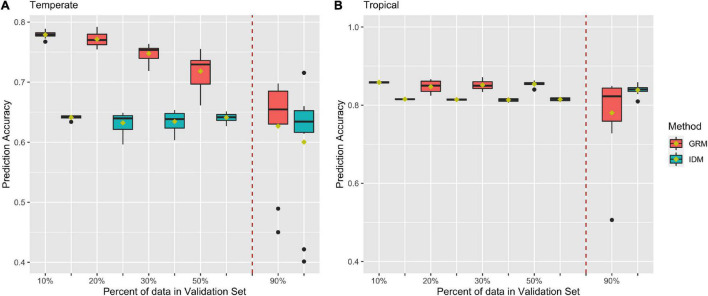
Prediction accuracy for black layer water content (BWC) in temperate **(A)** and tropical **(B)** ecological zones from the CV2) scenario (left of the dashed line) and an CV_90 scenario where 90% of the inbred lines were selected and had BWC phenotypes set to missing (right of the dashed line). Methods “GRM” and “IDM” refer to multi-trait models using a genomic relation matrix or identity matrix for the genetic effect, respectively.

Figures 5A,B shows prediction accuracies for BWC from CV2 in the temperate and tropical ecological zones, respectively. Red and blue box plots represent prediction accuracies from the MT-GBLUP model with genomic relationship matrix (GRM) or identity matrix (IDM), respectively. The box plots on left side of the dotted line are the prediction accuracies from 10-, 5-, 3-, and 2-fold cross-validation scenario. The box plots on right side of the dotted line are the prediction accuracies when 90% of the lines in the population were used as the validation set (CV_90). As shown in Figure 5A, prediction accuracies in temperate ecological zone range from 0.45 to 0.79 when the GRM was fit in the MT-GBLUP model, as compared to accuracies ranging from 0.4 to 0.65 when the identity matrix was used. Figure 5B shows prediction accuracies for BWC in the tropical ecological zone following the CV2 and CV_90 scenarios. When the GRM was fit in the MT-GBLUP model, prediction accuracies ranged from 0.5 to 0.87 as compared to accuracies ranging from 0.8 to 0.82 when the identity matrix was used. In general, the results from CV2 indicate that higher prediction accuracies are obtained when the GRM is used instead of an identity matrix in the MT-BLUP model.

Table 3 shows prediction accuracies in the temperate ecological zone following the CV3 scenario. As shown in Table 3, high prediction accuracies ranging from 0.79 to 0.96 were obtained for BWC in the temperate ecological zone from the MT-GBLUP model. This result indicates that unreplicated designs for BWC data collection can produce accurate results, with potentially large savings in labor and logistical costs.

**TABLE 3:** Prediction accuracy from the CV3 scenario for the temperate zone.

	Set-to missing	Accuracy
Replicate	Replicate 1	0.91
	Replicate 2	0.79
Location	Shenfu	0.86
	Shenyang	0.79
Replicate-location	Replicate 1—Shenfu	0.96
	Replicate 1—Shenyang	0.96
	Replicate 2—Shenfu	0.94
	Replicate 2–Shenyang	0.92

## Discussion

Development of maize varieties with low HWC is an ideal situation that ensures efficient mechanical harvesting can be applied. To achieve this goal, a good breeding strategy which can reduce the consumption of resources while achieving desired rates of genetic gain for the target traits is essential. In this study, we consider the genetic architecture of dry-down-related traits and effective prediction strategies for genomic-enabled breeding, leveraging correlated traits (HWC and FT) that are relatively easy to phenotype (Tsuruta et al., 2011; Jia and Jannink, 2012; Guo et al., 2014; Okeke et al., 2017; Lozada and Carter, 2019). Compared with single-trait genomic prediction model (ST-GBLUP), when a target trait has lower heritability and phenotypic data on highly correlated traits are available, multi-trait genomic prediction model (MT-GBLUP) has a great advantage (Guo et al., 2014). In MT-GBLUP, secondary traits are used to predict a target trait, which is often difficult to phenotype or measure (Lozada and Carter, 2019). The use of MT-GBLUP in US Holstein breeding efforts has improved prediction accuracy of several traits to varying degrees when compared to ST-GBLUP, (Tsuruta et al., 2011). Analogously, when multi-trait and multi-environment mixed models were used to predict agronomic traits, 40% improvement were obtain in prediction ability in cassava (Okeke et al., 2017).

The advantage of MT-GBLUP model, however, depends on the genetic correlation between the target and the secondary traits (Jia and Jannink, 2012). Estimates of (co) variance components for maturity and dry-down traits indicate that moderate-to-strong genetic correlations exist between routinely measured maturity and harvest moisture traits and the more difficult to measure traits like BWC and DR (Table 2). These results, combined with the moderate-to-low heritability found for DR (Table 1), suggest that genomic-enabled breeding strategies for selection on DR related traits should consider the use of correlated traits. The moderate-to-high correlations between HWC and BWC also indicate that strategies focused primarily on selection for HWC and yield could be used effectively to apply indirect selection pressure on DR. In this study, both hold (CV1 and CV2) and instant (CV_90) prediction accuracy calculations were used. It should be noted that the use of hold prediction accuracies can create negative bias in the correlations used to estimate prediction accuracy, this bias increases as the number of folds increases (Zhou et al., 2016).

The first cross-validation scenario (CV1) focused on breeding strategies that rely on generating predictions for lines that have yet to be tested in the field. As such, no phenotypic information on correlated traits is available on lines in the validation or prediction set. As seen in Figure 4, in situations where no correlated traits have been measured, MT-GBLUP model has no comparative advantage over ST-GBLUP when the training dataset has complete records for BWC. In fact, the results suggest that in this scenario, the use of ST-GBLUP may be a more parsimonious model leading to results that are as good or slightly better than MT-GBLUP. These results agree with previous findings that the advantage of MT-GBLUP is largest when the correlated traits were measured on prediction candidates and included in the model (Maier et al., 2015; Mehrban et al., 2019).

In the first cross-validation scenario (CV1), we further investigated the impact of including records in the training set that have no BWC phenotypic information but do have phenotypic records for correlated traits like FT and HWC. Many maize breeding programs record HWC and FT as agronomic traits for many generations (Abadassi, 2015), while BWC is rarely phenotyped. As a result, it is likely that historical data will have far more phenotypic data points for HWC and FT than for BWC. Historical HWC and FT provide breeders a considerable amount of historical data on correlated traits that can be used to predict BWC. The impact of including historical records without the target trait measured varies depending on how unbalanced the historical data are, but as the number of BWC records in the training set decreases, the advantage of MT-GBLUP increases when all correlated trait records are included for model training. These results suggest that including correlated traits in the training set can improve prediction accuracy substantially when there is sparse information on the target trait in historical datasets.

In CV2, the phenotype for BWC was set to missing for a subset of the population in order to mimic a breeding program that collects data on a trait that is expensive and difficult to measure on a subset of the population and predicts the phenotype for the rest of the population using routinely collected data on correlated traits. This is not an uncommon scenario as in most breeding programs, resource and time efficiency are important factors to consider (Morris and Bellon, 2004; Ceccarelli, 2015). The results in Figure 5 indicate that by using MT-GBLUP, BWC can be predicted with high accuracy for the majority of the population, thus reducing the cost and time that is required to record BWC for all lines being tested in the program.

As shown in Figure 5, the inclusion of correlated traits in the validation set resulted in significantly higher prediction accuracies (0.77 compare to 0.55) when compared to results from CV1, as the model exploits genetic correlation with the traits for which phenotypic data is available (Calus and Veerkamp, 2011; Lyra et al., 2017; Lado et al., 2018; Lozada and Carter, 2019; Runcie and Cheng, 2019). Prediction accuracies decreased as the number of lines with missing BWC data increased. Comparison of MT-GBLUP using a GRM with MT-GBLUP using the identity matrix shows that the GRM contributes significantly to prediction accuracy when there is more training data available for BWC. As the number of BWC phenotypic records decreases, the relative advantage of using the GRM decreases, indicating that the prediction accuracy is derived largely from correlated traits measured on the lines with missing BWC phenotypes when there are few BWC phenotypic records available to train the model. This trend is more pronounced in the temperate environments as compared to the tropical environment. The presence of several low prediction accuracy outliers for CV_90 is not unexpected given only 10% of the records have BWC information. The composition of the training set for these outliers was examined, and no obvious cause (i.e., population structure) of the lower accuracies was detected.

Multi-location and multi-replication trails play an important role in agronomic research and plant breeding programs (Crossa, 1990). In such cases, phenotyping a trait that is difficult or expensive to measure, such as BWC, in one location or one replication and predicting the phenotype for the other locations/replicates using correlated traits represents a cost-effective testing strategy. The CV3 scenario examines a sparse phenotyping approach in which only one replicate is phenotyped for BWC, while FT and HWC are phenotyped on all plots. The results show high accuracies for BWC predictions (>0.79), indicating that sparse phenotyping approaches can be effectively used to reduce the cost of BWC phenotyping without making large sacrifices in BWC predictions. This approach could be applied in combination with CV2, in which a sparse phenotyping approach is used for field trails after an initial line selection is made based on predictions from a MT-GBLUP model trained using historical BWC records as well data on correlated traits.

## Conclusion

In this study, multi-trait genomic prediction was tested using different cross-validation scenarios to investigate prediction strategies for genomic-enabled breeding for dry-down-related traits in maize. The results clearly show that the use of correlated traits, like HWC and FT, and sparse phenotyping can yield high prediction accuracies while reducing the cost of extensively phenotyping for difficult to measure traits like BWC. While the sparse phenotyping approaches consistently yielded very high prediction accuracies, the need to phenotype selection candidates on correlated traits places limitations on gains that can be made by increasing selection intensity and reducing generation intervals. Examining strategies for predicting untested lines, the accuracy of model prediction drops substantially when compared to sparse phenotyping; however, this strategy does enable gains in response to selection through increased selection intensity and reductions in the generation interval. Regardless of the breeding strategy, the results of this study show clear advantages to using correlated traits when information on the target trait is sparse in historical datasets.

## Data Availability Statement

The phenotype data presented in the study are included in the article/Supplementary Material; The genotype data reported in this paper have been deposited in the Genome Variation Map (GVM) in Big Data Center, Beijing Institute of Genomics (BIG), Chinese Academy of Science, under accession numbers GVM000350 at http://bigd.big.ac.cn/gvm/getProjectDetail?project=GVM000350; Further inquiries can be directed to the corresponding author.

## Author Contributions

PN was involved in manuscript preparation, phenotypic data collection, statistical analysis, development of analysis code, visualization, revision, and check. KR and MA were involved in manuscript preparation, statistical analysis, visualization, revision, and check. SW and YR were involved in conceptualization, research design, revision, and check. DD and LL were involved in phenotypic data collection, revision, and check. NM and ML were involved in revision and check. All authors contributed to the article and approved the submitted version.

## Conflict of Interest

The authors declare that the research was conducted in the absence of any commercial or financial relationships that could be construed as a potential conflict of interest.

## Publisher’s Note

All claims expressed in this article are solely those of the authors and do not necessarily represent those of their affiliated organizations, or those of the publisher, the editors and the reviewers. Any product that may be evaluated in this article, or claim that may be made by its manufacturer, is not guaranteed or endorsed by the publisher.

## Footnotes

1
https://tassel.bitbucket.io/

2
https://www.vsni.co.uk/software/asreml

## Supplementary Material

Supplementary Figure 1 Example scatter plot for phenotype value against predict value for **(A)** CV1 (20% BWC set to missing), **(B)** CV2 (5-fold CV in the temperate ecological zone), and **(C)** the scenario in which 90% of the inbred lines were random selected and had BWC phenotypes set to missing (CV_90).

Supplementary Table 1 Information on the phenotype data of dry-down-related traits.
