# Supplementary figures and images for: Genomic Prediction Strategies for Dry-Down-Related Traits in Maize
Source: Front Plant Sci. 2022 Jun 30;13:930429. doi: 10.3389/fpls.2022.930429 (PMC9280646; doi:10.3389/fpls.2022.930429)

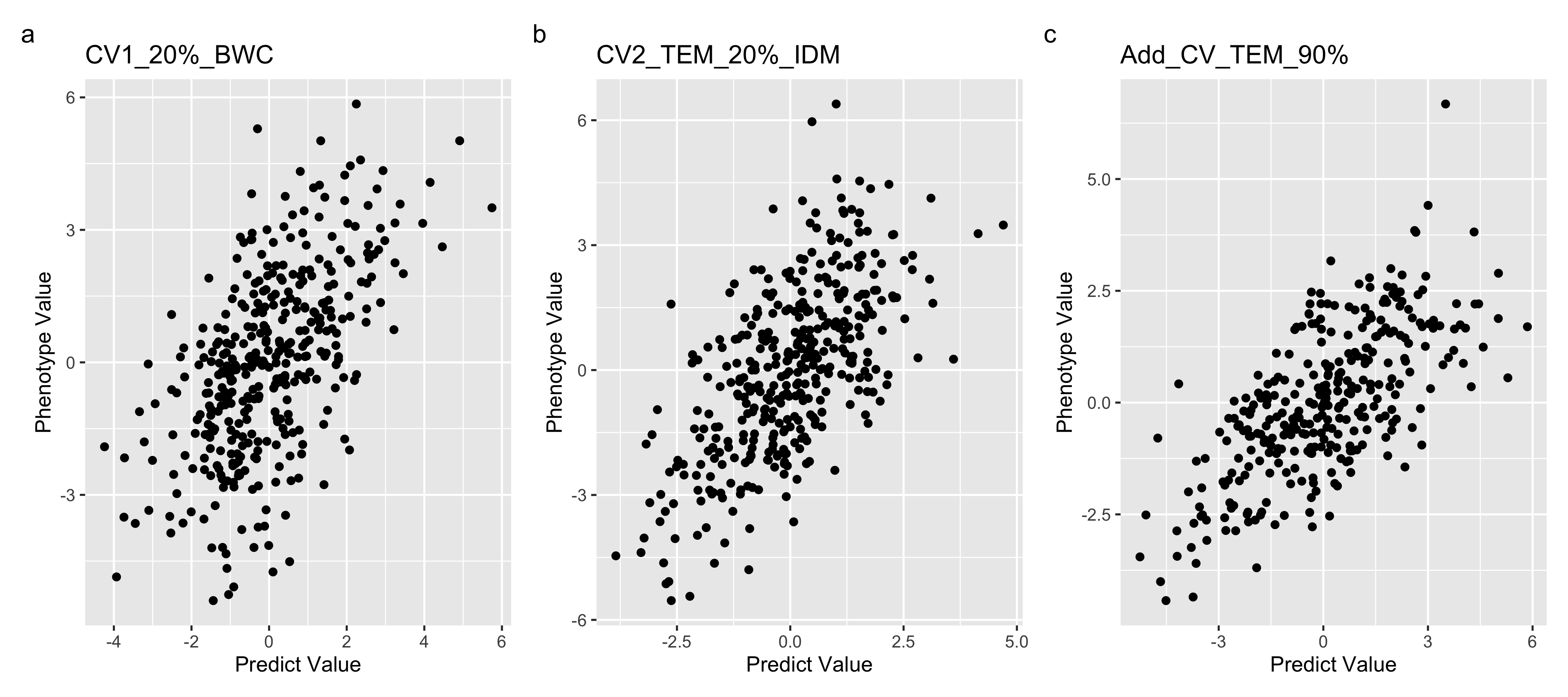

Supplement: Supplementary Figure 1 — Example scatter plot for phenotype value against predict value for (A) CV1 (20% BWC set to missing), (B) CV2 (5-fold CV in the temperate ecological zone), and (C) the scenario in which 90% of the inbred lines were random selected and had BWC phenotypes set to missing (CV_90). [file Image_1.JPEG]
